# Personal protective equipment-associated headaches in health care workers during COVID-19: A systematic review and meta-analysis

**DOI:** 10.3389/fpubh.2022.942046

**Published:** 2022-10-12

**Authors:** Ali Sahebi, Naser Hasheminejad, Masoumeh Shohani, Atefeh Yousefi, Somayeh Tahernejad, Azadeh Tahernejad

**Affiliations:** ^1^Non-Communicable Diseases Research Center, Ilam University of Medical Sciences, Ilam, Iran; ^2^Department of Occupational Health Engineering and Safety at Work, School of Public Health, Kerman University of Medical Sciences, Kerman, Iran; ^3^Department of Nursing, School of Nursing and Midwifery, Ilam University of Medical Sciences, Ilam, Iran; ^4^Department of Neurology, Shohadaye Tajrish Hospital, Shahid Beheshti University of Medical Science, Tehran, Iran; ^5^Health in Disasters and Emergencies Research Center, Institute for Futures Studies in Health, Kerman University of Medical Sciences, Kerman, Iran; ^6^Department of Health in Disasters and Emergencies, School of Public Health and Safety, Shahid Beheshti University of Medical Sciences, Tehran, Iran

**Keywords:** headache, personal protective equipment, health care workers, respiratory mask, COVID-19

## Abstract

**Introduction:**

Health Care Workers (HCWs) use Personal Protective Equipment (PPE) during the COVID-19 pandemic to protect themselves and prevent the transmission of the disease. The use of PPE, especially respiratory masks, has adverse consequences, including headaches, which have been secondary and unusual. The aim of the present systematic review and meta-analysis study was to investigate the prevalence of PPE-associated headaches in HCWs during COVID-19 pandemic.

**Methods:**

The present review study was performed based on the PRISMA guideline. The protocol of the present study was registered in PROSPERO with the code CRD42022304437. Valid data resources such as Scopus, PubMed, Web of Science, Science Direct, Google Scholar, Embase were used to identify and extract relevant studies. The searches were conducted between the beginning of 2020 and the end of January 2022. A random effects model was used for meta-analysis and *I*^2^ index was used to investigate between-study heterogeneity. Data were analyzed using STATA ver. 14.

**Results:**

A total of 539 articles were first identified through initial search and finally 26 final studies were selected to undergo the meta-analysis phase. According to the results of meta-analysis, the prevalence of headache after and before the use of PPE was 48.27% (95% CI: 40.20–56.34, *I*^2^ = 99.3%, *p* = 0 < 001) and 30.47% (95% CI: 20.47–40.47, *I*^2^ = 97.3%, *p* = 0 < 001), respectively.

**Conclusion:**

The results of the present study showed that the prevalence of PPE-associated headache in HCWs was relatively high, so, the use of PPE during COVID-19 pandemic can be considered as one of the causes of headache. Therefore, management strategies such as regular screening of HCWs for headaches and regular rest periods without the use of PPE can be effective in reducing the prevalence of headaches.

## Introduction

COVID-19 pandemic has affected the care activities of health care workers (HCWs) (1). Based on the available evidence, the Covid-19 virus is transmitted through close contact between individuals. Those who are in close contact with a Covid-19 patient or care for COVID-19 patients are at higher risk for the disease. HCWs are therefore required to use Personal Protective Equipment (PPE) to prevent the virus transmission while performing their duties to protect themselves (2–4).

According to the World Health Organization (WHO), PPE included guns, non-sterile gloves, goggles and respiratory masks (2). Although each country has its own certification standard for each mask type (3), in fact, the use of PPE by HCWs has unpleasant and annoying effects that will be exacerbated in the long run (4). However, long-term use of PPE is essential due to the prevalence of infectious diseases such as COVID-19 (4, 5).

The use of PPE, especially respiratory masks, can have a number of consequences, including headaches for HCWs. PPE-associated headaches are an unusual secondary headache, that mainly occur among HCWs due to the use of protective masks, Face masks and/or googles and have recently been studied in various studies (6, 7). PPE-associated headaches are considered as a subtype of external compression headaches (8). Although these headaches are often short-lived and without long-term side effects, they can affect occupational health, professional performance of HCWs, and their behavior in the proper use of PPEs (9).

Changes in staff conditions during the COVID-19 pandemic are more likely to cause PPE-associated headaches (6). Extended hours of work shifts (more than 8 h), combined use of PPE for long periods of time, or a higher physical and cognitive workload of HCWs when using PPE can increase the prevalence of PPE-associated headaches (7, 10–12). Studies have shown that using PPE every hour increases the risk of new symptoms (including headaches) by 1.38 times (18). Considering that the COVID-19 pandemic has provided an opportunity to study PPE-associated headaches among HCWs, the prevalence of PPE- associated headaches among HCWs has been assessed and reported in many studies since the onset of the COVID-19 pandemic (6, 13, 14). However, the results showed that there has been no comprehensive study on the prevalence of PPE- associated headaches HCWs during COVID-19 pandemic.

Therefore, this study was conducted with the aim of investigating the prevalence of headache associated with PPE in HCWs during COVID-19, and the prevalence of headache after using PPE and before using PPE was investigated among the studies conducted in this field. The results of this study can show whether HCWs are more prone to headaches after using PPE. Considering the importance of the subject matter, the results of the present systematic review and meta-analysis may provide an important source of information for health planning in addition to adding information about PPE-related headaches.

## Methods

Preferred Reporting Items for Systematic Reviews and Meta-Analyzes (PRISMA) guideline was used to conduct the present systematic review and meta-analysis (15). The protocol of the present review study was registered in International Prospective Register of Systematic Review (PROSPERO) with the code CRD42022304437.

### Data resources and search strategy

In this research, Data resources including PubMed, Scopus, Web of Science, Science Direct, Google Scholar, Embase, were used to search and extract studies. Also, valid keywords such as Headache^*^, “Head Pain^*^,” Cephalgia^*^, COVID 19, “SARS-CoV-2 Infection”, “SARS CoV 2 Infection,^*^” “2019 Novel Coronavirus Disease,” “2019 Novel Coronavirus Infection,” “2019-nCoV Disease,^*^” “COVID-19 Virus Infection,^*^” “Coronavirus Disease 2019,” “Coronavirus Disease-19,” “Coronavirus Disease 19,” “Severe Acute Respiratory Syndrome Coronavirus 2 Infection,” “SARS Coronavirus 2 Infection,” “COVID-19 Virus Disease,^*^” “2019-nCoV Infection,^*^” COVID19 OR “COVID-19 Pandemic,^*^” “COVID 19 Pandemic,” “Personal Protective Equipment,^*^” “PPE Personal Protective Equipment,” PPE OR Mask,^*^ “face shield,^*^” “Air-Purifying Respirator,^*^” goggle^*^, “Health Personnel,” “Health Care Provider,^*^” “Healthcare Provider,^*^” “Healthcare Worker,^*^” “Health Care Professional,^*^” “healthcare personnel,” “health care personnel,” “Medical Staff,” “Medical worker,” Search fields and operators were used to formulate the search strategy. The searches were conducted in English from the beginning of 2020 to the end of January 2022. The search strategy for types of databases is listed in Table 1.

**Table 1:** Lists the search strategies in various databases.

**Data base**	**Search strategy**
Pubmed	(Headache* OR “Head Pain*” OR Cephalgia*) AND (COVID 19 OR “SARS-CoV-2 Infection” OR “SARS CoV 2 Infection*” OR “2019 Novel Coronavirus Disease” OR “2019 Novel Coronavirus Infection” OR “2019-nCoV Disease*” OR “COVID-19 Virus Infection*” OR “Coronavirus Disease 2019” OR “Coronavirus Disease-19” OR “Coronavirus Disease 19” OR “Severe Acute Respiratory Syndrome Coronavirus 2 Infection” OR “SARS Coronavirus 2 Infection” OR “COVID-19 Virus Disease*” OR “2019-nCoV Infection*” OR COVID19 OR “COVID-19 Pandemic*” OR “COVID 19 Pandemic”) AND (“Personal Protective Equipment*” OR “PPE Personal Protective Equipment” OR PPE OR Mask* OR “face shield*” OR “Air-Purifying Respirator*” OR goggle*) AND (“Health Personnel” OR “Health Care Provider*” OR “Healthcare Provider*” OR “Healthcare Worker*” OR “Health Care Professional*” OR “healthcare personnel” OR “health care personnel” OR “Medical Staff” OR “Medical worker”)
Scopus	((ALL(Headache*) OR ALL(“Head Pain*”) OR ALL(Cephalgia*)) AND (ALL(COVID 19) OR ALL(“SARS-CoV-2 Infection”) OR ALL(“SARS CoV 2 Infection*”) OR ALL(“2019 Novel Coronavirus Disease”) OR ALL(“2019 Novel Coronavirus Infection”) OR ALL(“2019-nCoV Disease*”) OR ALL(“COVID-19 Virus Infection*”) OR ALL(“Coronavirus Disease 2019”) OR ALL(“Coronavirus Disease-19”) OR ALL(“Coronavirus Disease 19”) OR ALL(“Severe Acute Respiratory Syndrome Coronavirus 2 Infection”) OR ALL(“SARS Coronavirus 2 Infection”) OR ALL(“COVID-19 Virus Disease*”) OR ALL(“2019-nCoV Infection*”) OR ALL(COVID19) OR ALL(“COVID-19 Pandemic*”) OR ALL(“COVID 19 Pandemic”)) AND (ALL(“Personal Protective Equipment*”) OR ALL(“PPE Personal Protective Equipment”) OR ALL(PPE) OR ALL(Mask*) OR ALL(“face shield*”) OR ALL(“Air-Purifying Respirator*”) OR ALL(goggle*)) AND (ALL(“Health Personnel”) OR ALL(“Health Care Provider*”) OR ALL(“Healthcare Provider*”) OR ALL(“Healthcare Worker*”) OR ALL(“Health Care Professional*”) OR ALL(“healthcare personnel”) OR ALL(“health care personnel”) OR ALL(“Medical Staff”) OR ALL(“Medical worker”)))
Web of Science	((TS=(Headache*) OR TS= (“Head Pain*”) OR TS= (Cephalgia*)) AND (TS= (COVID 19) OR TS= (“SARS-CoV-2 Infection”) OR TS= (“SARS CoV 2 Infection*”) OR TS= (“2019 Novel Coronavirus Disease”) OR TS= (“2019 Novel Coronavirus Infection”) OR TS= (“2019-nCoV Disease*”) OR TS= (“COVID-19 Virus Infection*”) OR TS= (“Coronavirus Disease 2019”) OR TS= (“Coronavirus Disease-19”) OR TS= (“Coronavirus Disease 19”) OR TS= (“Severe Acute Respiratory Syndrome Coronavirus 2 Infection”) OR TS= (“SARS Coronavirus 2 Infection”) OR TS= (“COVID-19 Virus Disease*”) OR TS= (“2019-nCoV Infection*”) OR TS= (COVID19) OR TS= (“COVID-19 Pandemic*”) OR TS= (“COVID 19 Pandemic”)) AND (TS= (“Personal Protective Equipment*”) OR TS= (“PPE Personal Protective Equipment”) OR TS= (PPE) OR TS=(Mask*) OR TS=(“face shield*”) OR TS=(“Air-Purifying Respirator*”) OR TS=(goggle*)) AND (TS= (“Health Personnel”) OR TS= (“Health Care Provider*”) OR TS= (“Healthcare Provider*”) OR TS= (“Healthcare Worker*”) OR TS= (“Health Care Professional*”) OR TS= (“healthcare personnel”) OR TS= (“health care personnel”) OR TS= (“Medical Staff”) OR TS= (“Medical worker”)))

### Inclusion criteria

The inclusion criteria included English articles that investigated PPE-associated headaches in HCWs during COVID-19 pandemic.

### Exclusion criteria

Case report studies, review studies, intervention studies, letter-to-the editor and headache report in non-HCWs as well as report of PPE-associated headache in HCWs in non-COVID 19 situation and also during other pandemics.

### Selection of studies

Endnote 7 software was used to collect the primary identified studies. After removing the duplicates, the titles and abstracts of the remaining studies were screened. In the studies selection phase, two researchers independently read the full text of potentially relevant studies and finally selected studies for qualitative assessment. Any disagreement between reviewers was resolved by a third reviewer.

### Qualitative assessment and data extraction

After selecting the studies, two researchers independently used the Tool Appraisal tool for Cross-Sectional Studies (AXIS) (16) to assess the quality of the selected studies. The possible score range is 0 to 20 and studies with a score above 12 entered the meta-analysis phase. Any disagreement between the reviewers was resolved by the third reviewer. Also, in the data extraction stage, two researchers independently extracted the type of PPE used as well as the prevalence of subsequent headache before and after the use of PPE and using a pre-prepared checklist that includes information on first author, place of study, average age, number of men and women, sample size, duration of PPE use. A third person was used to resolve any disagreement between the two researchers.

### Statistical analysis

I2 index was used to evaluate the between-study heterogeneity. *I*^2^ index <25%, 25–50%, 50–75% and more than 75% showed no heterogeneity, moderate, high and very high heterogeneity, respectively (17). In order to reduce the between-study heterogeneity, a random effects model was used for meta-analysis. Begg test was used to evaluate the publication bias. Data analysis was carried out using STATA software ver. 14.

## Results

**Figure 1:**
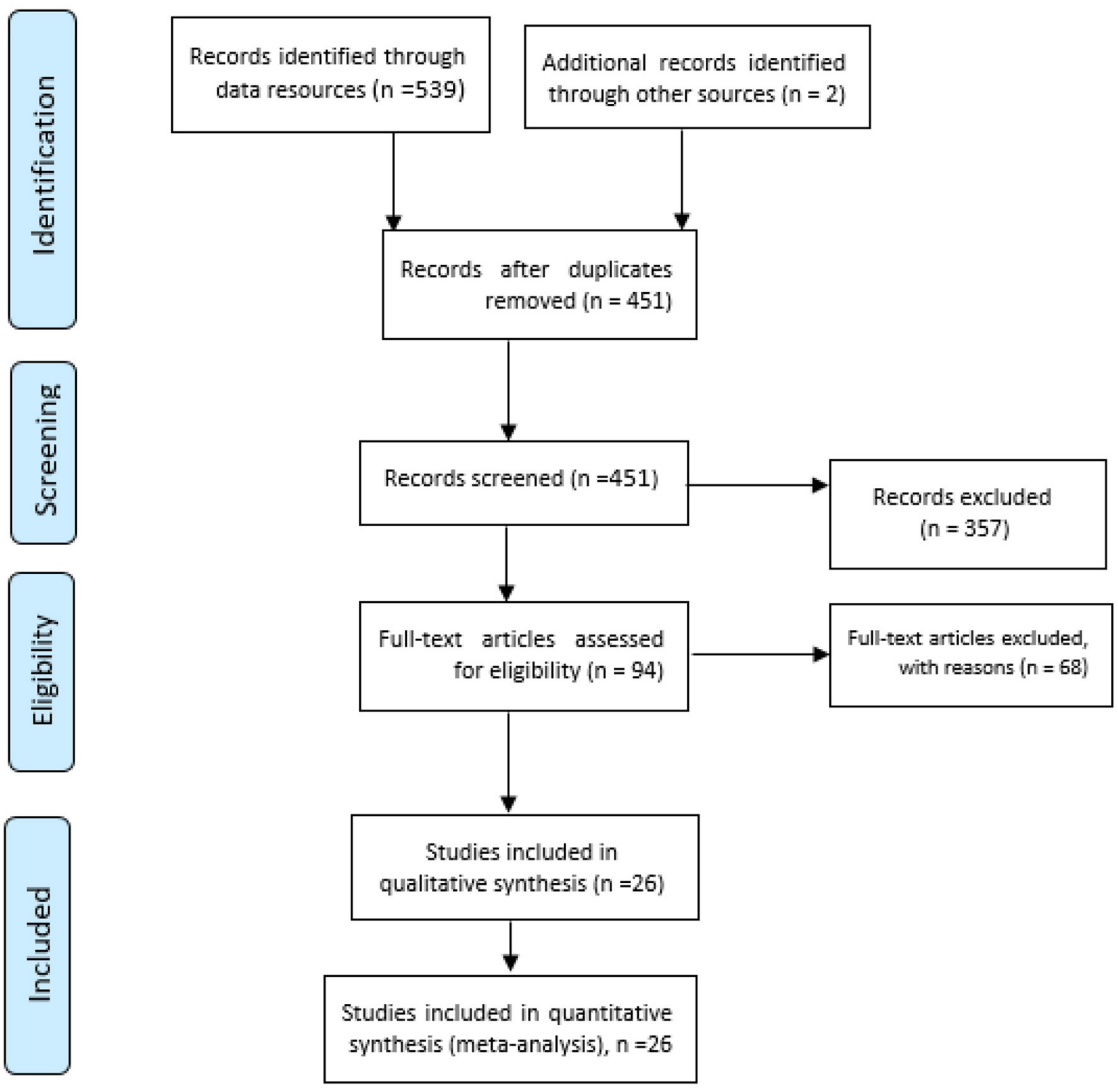
Flowchart of the selection of studies based on PRISMA.

In this review, 539 initial articles were identified by searching the aforementioned databases. After eliminating duplicates, 451 studies were screened. Afterwards, 94 studies were selected, and finally, 26 studies were selected to undergo quality assessment and then all of them entered the meta-analysis phase (Figure 1). Also, 14,172 HCWs were examined for headaches, of whom 7,986 were male and 6,186 were female. All studies were cross-sectional studies (Table 2). Among the selected studies, face shields were used in 5 studies and protective glasses were used along with masks in 4 studies. In most of the studied studies, surgical masks were used, and in some studies, N95 masks were also used along with surgical masks (Table 2). It should be noted that in the selected studies, most of the study participants used PPE more than 4 h a day. More detailed information about the time of use of the participants in the reviewed studies is presented in Table 2.

**Table 2:** The characteristics of studies included in the meta-analysis.

**First author**	**Location**	**Sample size**	**Male**	**Female**	**Mean Age (SD)**	**Pre-existing headaches**	**Post use PPE headache**	**Type of PPE**	**Duration of PPE use**
Hajjij et al. (10)	United Arab Emirates	155	48	107	32 (9.32)	29%	32.9%	- N95 Mask - Surgical Mask - Eyes Protective equipment	<4 h (32.9%) >4 h (67.1%)
Ong et al. (6)	Singapore	158	47	111	-	29.1%	81%	- N95 Mask - Goggles - Face shield/visor	<4 h (16.46%) >4 h (83.54%)
Zaheer et al. (18)	Pakistan	241 51 (21.1)	128	113	28.5 (6.2)	21.1%	28.2%	- N95 Mask - Eyes Protective equipment	<4 h (10%) >4 h (90%)
Ramirez-Moreno et al. (19)	Spain	306	62	244	43	41.1%	51.6%	- surgical Mask - N95 Mask - Face shield - Protective eyewear	Mean (SD) = 6.9 (2.3) h
Rapisarda et al. (20)	Italy	383	134	249	33.4 (9.2)	56.65	26.5%	- Surgical Mask - Other mask	<4 h (6.3%) >4 h (93.7%)
Toksoy et al. (21)	Turkey	375	161	214	-	30.4%	30.9%	- Filtering Mask - Surgical Mask - Filtering + surgical Mask	<4 h (7.7%) >4 h (92.3%)
Jafari et al. (22)	Iran	243	61	182	36 (8)	44.3%	77%	-N95 Mask -surgical Mask -N99 Mask -Goggle -Face shield	>4 h
Joy et al. (23)	Bangladesh	200	129	71	35.4 (7.5)	11.1%	59.9%	- N95/FFP3/FFP2 Mask - surgical Mask - N99 Mask - Half/Full Respirator - Goggle -Face shield	<6 h (19.5%) >6 h (80.5%)
Çaglar et al. (13)	Turkey	315	156	159	31.5 (4.6)	-	36.5%	- N95/FFP2 Mask	>4 h
Jose et al. (24)	India	137	64	73	30.4 (3.3)	-	73.4%	N95 Mask	6 h
Thiagarajan et al. (25)	India	342	275	67	-	11.4%	43%	-N95 Mask - Face shield	<3 h (31%) >3 h (62.3%)
Hacibeyoglu et al. (14)	Turkey	177	103	74	32.3 (7.3)	31.1%	65.5%	- N95/FFP2 Mask - Surgical Mask	<4 h (6.2%) >4 h (93.8%)
Tabah et al. (26)	Australia	2,711	1,457	1,254	41	-	28%	-N95/FFP2/FFP3 Mask - Surgical Mask PAPR	Median = 4 h
Bansal et al. (27)	India	309	146	163	-	-	44%	-N95 Mask -Surgical Mask -Protective goggle	<4 h (12.9%) >4 h (87.1%)
Davey et al. (28)	UK	224	32	192	-	-	79%	-N95/N99/FFP2/ FFP3 Mask - Surgical Mask	<4 h (26.8%) >4 h (73.2%)
Çiriş Yildiz et al. (29)	Turkey	553	166	387	-	-	74.1%	-N95/FFP2 Mask -Surgical Mask -protective glasses	-
Radhakrishnan et al. (30)	India	2,451	1,737	714	-	-	0.096%	Surgical Mask	-
Shubhanshu et al. (31)	India	423	320	103	-	-	23%	-N95 Mask - Surgical Mask	>4 h
Bharatendu et al. (32)	Singapore	154	51	103	29 (12)	-	79.9%	N95 Mask	-
Rosner et al. (33)	USA	343	28	315	-	-	71.4%	-N95 Mask - Surgical Mask	>4 h
Bai et al. (34)	Pakistan	126	104	22	40.9 (7.31)	-	69.2%	face Mask	<6 h (25.4%) >6 h (74.6%)
Agarwal et al. (35)	India	253	-	-	42.1 (11)	-	28%	-N95 Mask - Surgical Mask	<4 h (28%) >4 h (72%)
Arif et al. (36)	Pakistan	196	96	100	-	-	62.5%	-N95 Mask - Surgical Mask	-
Peres et al. (37)	Portugal	3,052	500	2,550	-	-	37.5% 19.4%	N95 Mask	<4 h (1.6%) >4 h (98.4%)
Cigiloglu et al. (38)	Turkey	311	166	145	33.4 (7.0)	-	47.6%	Surgical Mask FFR Mask	Mean (SD) = 9.0 (2.0)
Ipek et al. (39)	Turkey	34	15	19	31.3 (6.3)	-	59% 15%	N95 Mask Surgical Mask	<4 h 100%

**Figure 2:**
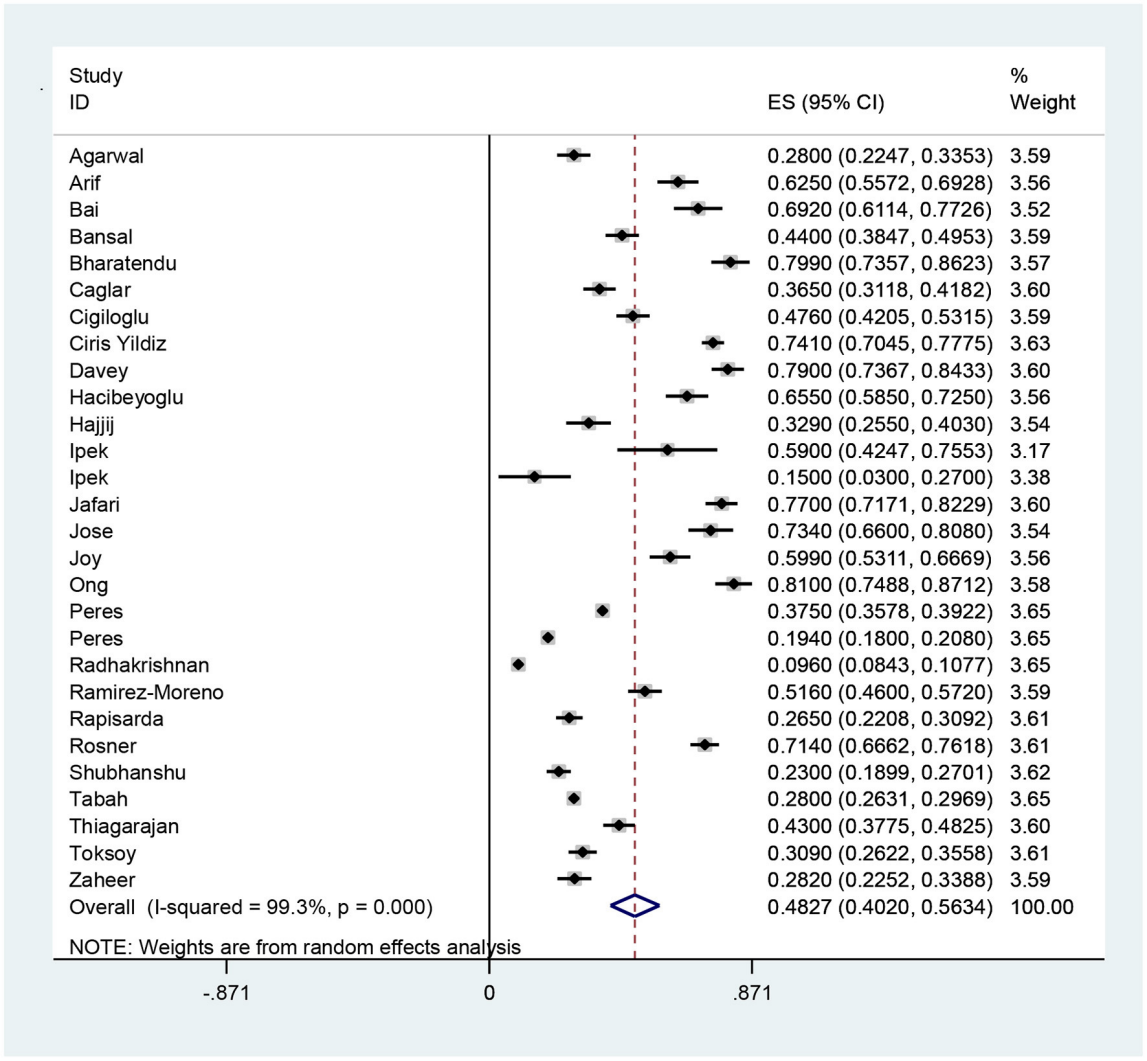
The prevalence of headache after the use of PPE and 95% confidence interval for each of the reviewed studies and the total studies. The midpoint of each segment shows the headache estimate and the length of the segment shows a 95% confidence interval. The diamond symbol indicates the total headache.

**Figure 3:**
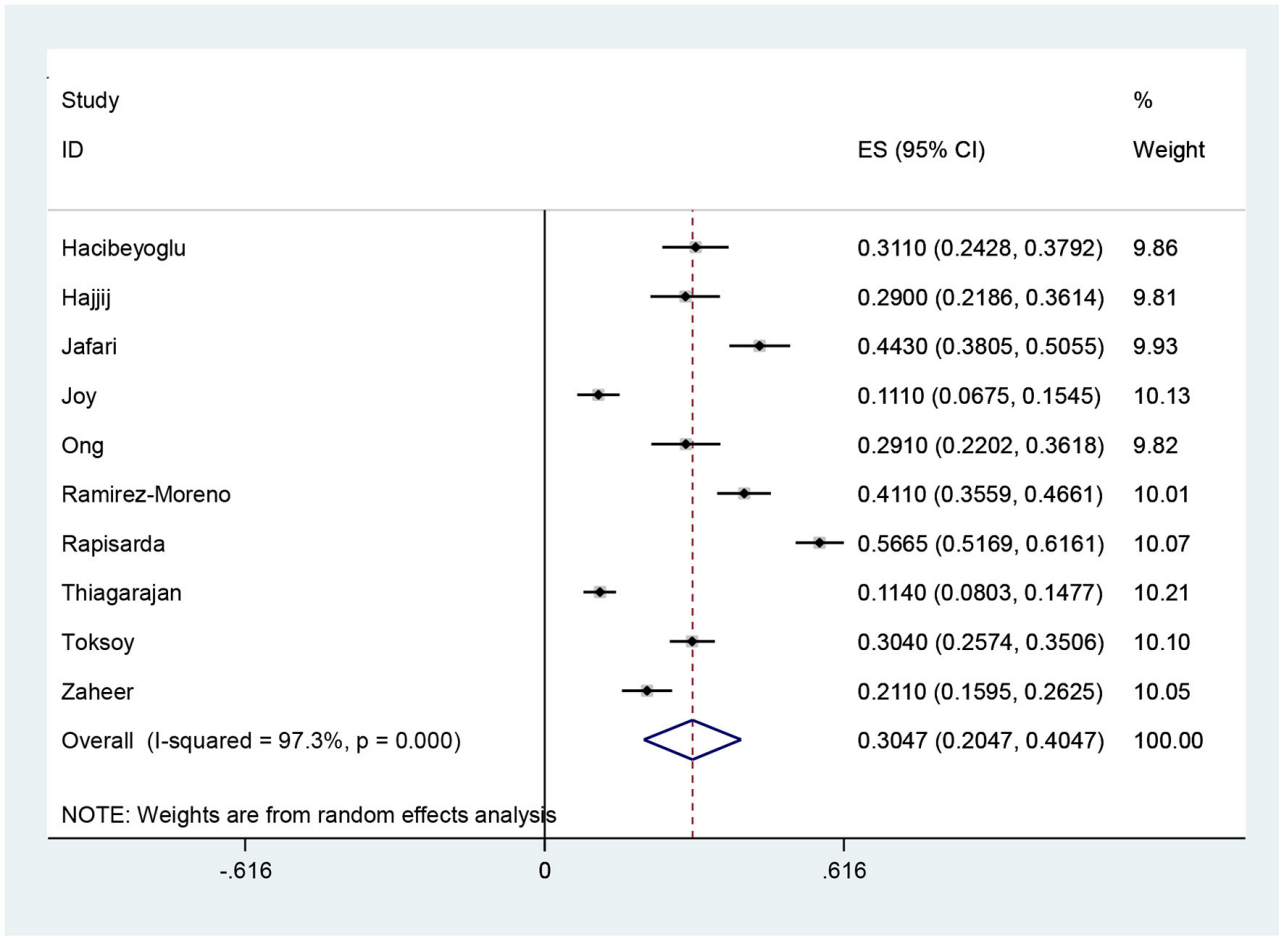
Headache rate before the use of PPE and 95% confidence interval for each of the reviewed studies and the total studies. The midpoint of each segment shows the headache estimate and the length of the segment shows a 95% confidence interval. The diamond symbol indicates the total headache.

**Figure 4:**
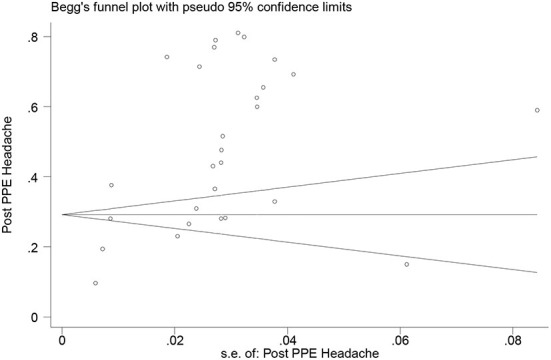
Bias publication based on Begg's test regarding headache after the use of PPE.

**Figure 5:**
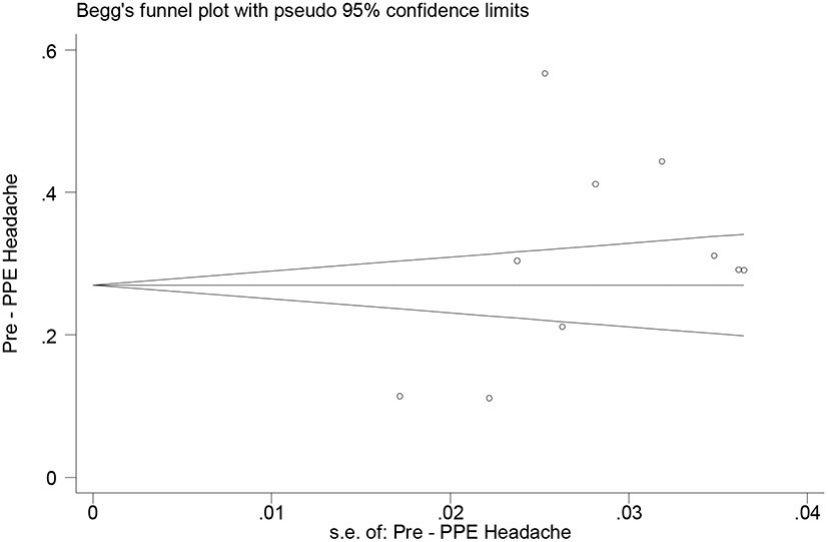
Bias publication based on Begg's test for headache before the use of PPE.

According to the results of meta-analysis, the prevalence of headache after and before the use of PPE was 48.27% (95% CI: 40.20–56.34, *I*^2^ = 99.3%, *p* = 0 < 001) (Figure 2) and 30.47% (95% CI: 20.47–40.47, *I*^2^ = 97.3%, *p* = 0 < 001), respectively (Figure 3). The *I*^2^ index showed that the between-study heterogeneity is very high. Results of Begg test showed that publication bias in headache after (*P* = 0.133) and before the use of PPE (*P* = 0.531) was negligible (Figures 4, 5).

## Discussion

In the present review study, which was performed to investigate the prevalence of PPE-associated headaches in HCWs during the COVID-19 pandemic, 26 studies were selected for meta-analysis. According to the results of the present meta-analysis, the prevalence of headache after and before the use of PPE use was 48.27% and 30.47%, respectively, indicating that HCWs are more prone to PPE after using it.

In a study on psychological consequences and physical symptoms of HCWs during COVID-19, Chew et al. (40) showed that the prevalence of headache among HCWs was 31.9% and that there is a significant relationship between psychological disorders and physical symptoms (40). In a study on depression among neurosurgeons during COVID-19 pandemic, Sharif et al. (41) reported a headache rate of 20% (41). Yifan et al. (42) reported a headache rate of 19.3% when examining nurses' physical disorders during the Covid-19 pandemic (42). Since the results of the previous studies are consistent with the present study and emphasize the prevalence of headaches without the use of PPE in HCWs, so, it is suggested that health managers should assess these people for headaches due to several factors, including physical and psychological factors. On the other hand, in this study, the prevalence of headache associated with the PPE use was higher than studies in the study of headache without the PPE use, which shows the significant effect of the PPE use on headache among HCWs during the COVID-19 pandemic.

In study on HCWs who used the N95 mask during the SARS epidemic, Lim et al. (43) reported that the prevalence of face mask-associated headaches was 37.3% (43), which is lower than in the present study. Given that the COVID-19 disease is a pandemic and has affected HCWs for a long time, this difference in the prevalence of headache among HCWs can be justified.

A review study investigated the physiological and adverse effects of PPE use and results showed that headache was significant among other side effects (44). In a report sent as a letter-to-the editor, Swaminathan et al., reported that the prevalence of PPE-associated headache was 61.7% (45). The results of these studies are also consistent with the present study.

The face mask and eyewear can exert mechanical forces and stimulate superficial sensory neurons in the skull and neck (46, 47). Scarano et al. showed that long-term use of the FFP2 mask reduced hemoglobin oxygen saturation, increased heart rate, and facial temperature, which may lead to stress and headaches among HCWs (48). High blood carbon dioxide levels can also contribute to side effects such as dizziness, shortness of breath and headache (47). In these studies, factors have been identified as possible risk factors for headaches that are likely to occur to HCWs with the use of PPEs during the COVID-19 pandemic. Therefore, the results of the present study were not unexpected.

Various studies have concluded that headache is associated with prolonged PPE use (13, 24). Spontaneous headache relief has been reported within 60 min after PPE removal (6). Considering that duration of PPE use has increased during the Covid-19 outbreak, it is obvious that, as the results of the present study showed, the prevalence of headache will also increase during this period.

The effect of headache on personal health (both physical and mental health) and work performance has been confirmed in various studies (49–51). Although various factors, such as increased psychological and physical overload and occupational psychosocial stressors (52), may have caused headaches among HCWs during the outbreak of COVID-19 (40, 41), the PPE use has been cited in various studies as a major cause of headache. In their review study, Ong et al. discussed the clinical features and possible causes of PPE-associated headaches during the COVID-19 pandemic (7). In a narrative review, Romero et al. investigated the causes of headaches among HCWs during COVID-19 and stated that the physical and homeostatic effects of PPE use may also affect headache (47).

Proper PPE use is crucial for protecting HCWs from COVID-19 and airborne-transmitted infections (53). Therefore, since the results of the present study indicate a significant prevalence of PPE-associated headaches, it is worthwhile for planning committees to take steps to minimize the negative effects of PPE on their personnel and to increase their efforts to address this issue.

However, it is remarkable that PPE-associated headaches are also associated with psychological stress, depression, and sleep disorders, which increased during COVID-19 outbreak among HCWs, as headache is also the most common COVID-19-related neurological symptom (43). Because the aggravation of headaches can have a great impact on the wellbeing and occupational performance of HCWs.

### Limitations

In this study, there was a high heterogeneity between studies, because in different studies that were conducted in this field, the sample size and tools used to assess the prevalence of PPE-Associated headaches were different.

We also failed to assess the prevalence of PPE- associated headaches by gender, Because most studies in this field have not done so. In addition, the timing of PPE use is likely to affect the prevalence of headache, but due to the fact that PPE timing varied in studies, we were unable to investigate this association.

## Conclusion

The present study shows that a significant increase in the prevalence of headache among HCWs during the COVID-19 pandemic. In addition, the PPE use could be one of the causes of headache among HCWs during the COVID-19 pandemic, because the prevalence of headache after the use of PPE was higher than the prevalence of headache before the use of PPE in the present study. Therefore, further studies are needed to investigate the effects of PPE use on the onset of different categories of headache and other neurological conditions. Also, considering the effect of headache on HCWs performance (especially cognitive performance), which can lead to medical errors, in order to achieve a better PPE-user fit, further studies should be performed to provide intervention strategies in order to improve ergonomic features of PPEs that are used for a long time. In addition, management strategies such as regular screenings of HCWs for headaches and devoting rest periods so that HCWs can avoid using PPE for a period of time may also be effective in reducing the incidence of headaches. Therefore, it is recommended to conduct further studies in this field and investigate different solutions.

## Author contributions

AS and ST managed the project. AS, ST, NH, and MS developed the inclusion criteria, screened titles, and abstracts. AY, AS, AT, and ST read and screened articles for inclusion. NH, AY, MS, and ST performed the qualitative assessment and data extraction. AS as a statistician and re-checked statistical analysis. ST, MS, and AT was a major contributor in writing the manuscript. All authors read and approved the final manuscript.

## Conflict of interest

The authors declare that the research was conducted in the absence of any commercial or financial relationships that could be construed as a potential conflict of interest.

## Publisher's note

All claims expressed in this article are solely those of the authors and do not necessarily represent those of their affiliated organizations, or those of the publisher, the editors and the reviewers. Any product that may be evaluated in this article, or claim that may be made by its manufacturer, is not guaranteed or endorsed by the publisher.
